# Proteomic Profiling of Early Pre-Ribosomal Complexes from Human Nucleolar Extracts

**DOI:** 10.32607/actanaturae.27928

**Published:** 2026

**Authors:** K. L. Dobrochaeva, D. K. Kovaleva, A. A. Moraleva, R. Kh. Ziganshin, A. S. Deryabin, Yu. P. Rubtsov

**Affiliations:** Shemyakin–Ovchinnikov Institute of Bioorganic Chemistry, Russian Academy of Sciences, Moscow, 117997 Russia; Faculty of Biology, Lomonosov Moscow State University, Moscow, 119234 Russia; Faculty of Chemistry, Lomonosov Moscow State University, Moscow, 119991 Russia

**Keywords:** nucleolus, ribosome biogenesis, nucleolar extraction, proteomic analysis

## Abstract

The nucleolus is a key cellular compartment responsible for the major steps of ribosomal subunit biogenesis. Detailed characterization of the nucleolar proteome is essential for understanding the molecular mechanisms underlying ribosome biogenesis and may help identify novel regulators of this process. However, the nucleolar proteome, and the protein composition of early ribosomal subunit precursors in particular, remain poorly defined, largely because of the methodological inconsistencies and conflicting findings across published studies. Here, we aimed to establish a standardized approach that could be used to identify novel factors involved in the early stages of ribosome biogenesis using an optimized stepwise nucleolar extraction protocol, followed by sucrose density gradient fractionation and mass spectrometry analysis. This optimized approach allowed us to enrich for proteins directly engaged in early biogenesis steps, while depleting non-specific nucleolar “background” components. A total of 529 unique proteins were identified across two biological replicates. Gene Ontology (GO) analysis revealed significant enrichment of proteins annotated as ribosome biogenesis factors, including those directly involved in ribosomal subunit assembly and rRNA processing. A cross-referencing of our dataset against 188 known components of pre-ribosomal complexes represented in the PDB yielded 84 overlapping proteins. Early biogenesis factors prevailed in this group (70 out of 84), supporting the idea of the enrichment of the analyzed fraction with early pre-ribosomal complexes. The resulting protein dataset will facilitate the identification of novel factors governing the poorly understood aspects of human ribosome biogenesis.

## INTRODUCTION

The nucleolus is a membraneless compartment of the cell nucleus in which the key stages of ribosome biogenesis occur: (1) transcription of ribosomal DNA (rDNA) genes; (2) processing of ribosomal RNA precursors (pre-rRNA); and (3) the assembly of pre-ribosomal particles. The nucleolus comprises three structural components: the fibrillar center, dense fibrillar component, and granular component. These compartments undergo dynamic reorganization, and their protein composition changes in response to the physiological state of the cell and to external stimuli [1, 2].

Despite substantial progress in characterizing the protein composition of the nucleolus, its full proteome remains incompletely defined, because different experimental strategies have yielded substantially different sets of proteins. For example, nucleolar isolation by sequential centrifugation of cell lysates in a sucrose density gradient [3] followed by mass spectrometry helped identify 4,500 different proteins according to the NOPdb database [4]. In another study, only 1,318 nucleolar proteins were confirmed by immunofluorescence confocal microscopy [5]. A recently published integrated dataset combining NOPdb and Gene Ontology records with the findings of Orre et al. identified 3,490 nucleolar proteins [6]. This lack of consistency emphasizes the fact that the nucleolar proteome composition depends on the isolation method and criteria for protein inclusion, as well as on the type and functional state of cells.

Nucleolar proteome characterization is especially important when studying ribosome biogenesis in humans, since the organization and regulation of this process are more intricate than they are in model organisms, particularly *Saccharomyces cerevisiae*. Over 200 factors involved in the regulation of the assembly of ribosomal subunits, in coordination with numerous small nucleolar RNAs, have been identified in yeast. By contrast, recent studies of the mechanisms orchestrating human ribosome biogenesis confirm that human cells rely on additional regulation levels and an expanded repertoire of assembly and quality control factors. Moreover, the regulation of ribosome biogenesis is not confined solely to the static repertoire of resident nucleolar proteins: functional screening, including high-throughput analysis of microRNA mimic libraries, has revealed previously unknown repressors of ribosome biogenesis, indicating the existence of additional regulatory pathways that modulate nucleolar processes [7, 8, 9].

However, an analysis of the entire nucleolar proteome does not always allow one to distinguish between proteins that are intrinsic components of early pre-ribosomal complexes and the broader range of resident nucleolar components. Therefore, to be able to identify novel players in ribosome biogenesis, it is essential to employ approaches that enrich fractions containing sedimenting pre-ribosomal particles, rather than comprehensively profiling the entire nucleolar proteome. In this study, we performed sucrose gradient fractionation of the nucleolar extract, followed by mass spectrometry analysis of pooled fractions, thereby focusing our analysis on sedimenting nucleolar fractions enriched in early pre-ribosomal particles and narrowing the search for potential novel factors involved in their maturation.

## EXPERIMENTAL

Human HEK293T cells (Russian Collection of Cell Cultures, Institute of Cytology, Russian Academy of Sciences) were cultured in DMEM (PanEco, Russia) supplemented with 10% fetal bovine serum (Hyclone, UK) at 37°C in an atmosphere of 5% CO_2_.

For sucrose gradient centrifugation, the cells were cultivated in ten T75 cell culture flasks to ~ 80% confluence (a total of ~ 2 × 10^8^ cells) under standard conditions, detached using a 0.05% trypsin solution and 0.5 mM EDTA, washed with phosphate-buffered saline, frozen in liquid nitrogen, and stored at –80°C until use. The cell pellet was thawed on ice, and the nuclei were extracted using the protocols described in refs. [3, 10]. The purified cell nuclei were consecutively treated with buffers for nucleolar fractionation (buffer 1: 10 mM HEPES, 10 mM NaCl, 5 mM MgCl_2_, 0.1% Igepal CA-630, 0.5 mg/mL heparin, and 600 U/mL RNase inhibitor pH 7.5; buffer 2: 20 mM HEPES-NaOH pH 7.5, 200 mM NaCl, 4 mM EDTA, 0.1% Igepal CA-630, 0.04% sodium deoxycholate, 4 mM imidazole, 0.1 mg/mL heparin, 1 mM DTT, protease inhibitor, and 600 U/mL RNase inhibitor). The isolated fractions were centrifuged at 12,300 *g* for 10 min at 4°C. The resulting supernatants (two biological and two technical replicates) were applied onto a linear 10–50% sucrose gradient in a buffer containing 20 mM HEPES-NaOH (pH 7.5), 200 mM NaCl, 4 mM EDTA, 0.1% Igepal CA-630, 0.1 mg/mL heparin, and 1 mM DTT. Ultracentrifugation was conducted using an SW40Ti rotor (Beckman Coulter, USA) at 36,000 rpm for 4 h at 4°C. After the centrifugation, the density gradients were manually fractionated into 0.5 mL fractions; the optical density of each specimen at 260 nm was measured, and the proteins were extracted.

The purified protein samples were incubated in Laemmli buffer (10 min, 100°C) and separated by SDS–PAGE on a 12% polyacrylamide gel. The proteins were transferred onto a nitrocellulose membrane with a pore size of 0.2 µm (Bio-Rad, USA). The membrane was blocked in 5% skim milk in TBST (20 mM Tris-HCl, 150 mM NaCl, 0.1% Tween 20 pH 7.6) during 1 h at room temperature and incubated with primary antibodies against the RPS10 and RPL19 proteins.

Reduction, alkylation, and digestion of the proteins were performed as described previously [1], with minor modifications. Briefly, 20 μl of sodium deoxycholate (SDC) reduction and alkylation buffer pH 8.5 containing 100 mM TRIS, 1% (w/v) SDC, 10 mM TCEP, and 20 mM 2-chloroacetamide were added to a 10 μg protein sample. The sample was sonicated in an ultrasonic water bath for 1 min, heated at 85°C for 10 min, cooled to room temperature, and an equal volume of a trypsin solution in 100 mM TRIS pH 8.5 was added at a 1 : 50 (w/w) ratio. After overnight digestion at 37°C, peptides were acidified by 50 μl of 2% trifluoroacetic acid (TFA), mixed with 50 μl of ethyl acetate, and loaded on SDB-RPS StageTips equipped with two 14-gauge SDB-RPS plugs. The StageTip was centrifuged at 300 *g* until the entire solution had passed through the StageTip (typically 4 min). After washing the StageTips with 100 μl of the 1% TFA/ethyl acetate 1 : 1 mixture (2 times) and 50 μl of 0.2% TFA, the peptides were eluted in a clean tube by 60 μl of a 50% acetonitrile/5% ammonia mixture using centrifugation at 300 *g*. The collected material was vacuum-dried and stored at –80°C. Before the analyses, the peptides were dissolved in 20 μl of 2% acetonitrile/0.1% TFA [11].

Samples were loaded onto a home-made trap column 50 × 0.1 mm in size, packed with Reprosil-Pur 200 C18-AQ 5 μm (Dr. Maisch) and in the loading buffer (2% ACN, 98% H_2_O, 0.1% TFA) at a 4 μl/min flow rate and separated at RT in a home-packed [1] fused-silica column 300 × 0.1 mm in size packed with Reprosil-Pur C18-AQ 1.9 μm (Dr. Maisch) into an emitter prepared with the P2000 Laser Puller (Sutter, USA). Reverse-phase chromatography was performed with an Ultimate 3000 Nano LC System (Thermo Fisher Scientific), which was coupled to a Orbitrap Tribrid Ldumos mass spectrometer (Thermo Fisher Scientific) via a nanoelectrospray source (Thermo Fisher Scientific). Water containing 0.1% (v/v) FA was used as mobile phase A and ACN containing 0.1% FA (v/v), 20% (v/v) and H_2_O as mobile phase B. The peptides were eluted from the trap column with a linear gradient: 3% B for 5 min; 3–25% B for 28 min, 25–40% B for 25 min, 40–60% B for 4 min, 60% B during 3 min, 60–99% B for 0.1 min, 99% B during 10 min, and 99–2% B for 0.1 min at a flow rate of 500 nl/min. MS data was collected in DDA mode. MS1 parameters were as follows: 60K resolution, 350–1600 scan range, max injection time – auto, AGC target – standard. Ions were isolated with a 1.2 m/z window, a preferred peptide match, and isotope exclusion. Dynamic exclusion was set to 20 s. MS2 fragmentation was conducted in the HCD mode at 15K resolution with HCD collision energy of 30%; max injection time – 80 msec; and AGC target – standard. Other settings: charge exclusion – unassigned; 1, > 6 [12].

MS raw files were analyzed using Peaks studio 11 (Bioinformatics Solutions Inc.) [1]. Identification of proteins was achieved by searching against the databases for *Homo sapiens* (Uniprot-SwissProt version of 05.2023) with a carbamidomethyl Cys as a fixed modification, acetylation (N-term), deamidation Asn/Gln, and Met oxidation as real modifications. The false discovery rate for peptide-spectrum matches was determined by searching a reverse database and was set to 0.01. Enzyme specificity was set as C-terminal to arginine and lysine (semi-specific), and a maximum of three missed cleavages were allowed in the database search. Peptide identification was performed with an allowed initial precursor mass deviation of up to 10 ppm and an allowed fragment mass deviation of 0.02 Da [13].

The data were analyzed using Microsoft Excel and the gProfiler web-based toolset.

## RESULTS AND DISCUSSION

The maturation of early pre-ribosomal complexes in the nucleolus of human cells is the least studied ribosome biogenesis stage. We hypothesized that previously undescribed ribosome biogenesis factors could be identified by mass spectrometry analysis of sucrose gradient fractions containing early pre-ribosomal particles, rather than by the analysis of the entire nucleolar proteome, given its highly dynamic composition. The nucleolar extracts obtained using the adapted protocol proposed by Nieto et al. [10] were fractionated by sucrose gradient ultracentrifugation and analyzed using Western blotting (*Fig. 1*). Our findings show that ribosomal protein signals were distributed unevenly across the gradient and peaks enriched in regions corresponding to characteristic peaks in the gradient profile, thus confirming that the analyzed samples contained pre-ribosomal particles.

**Fig. 1:**
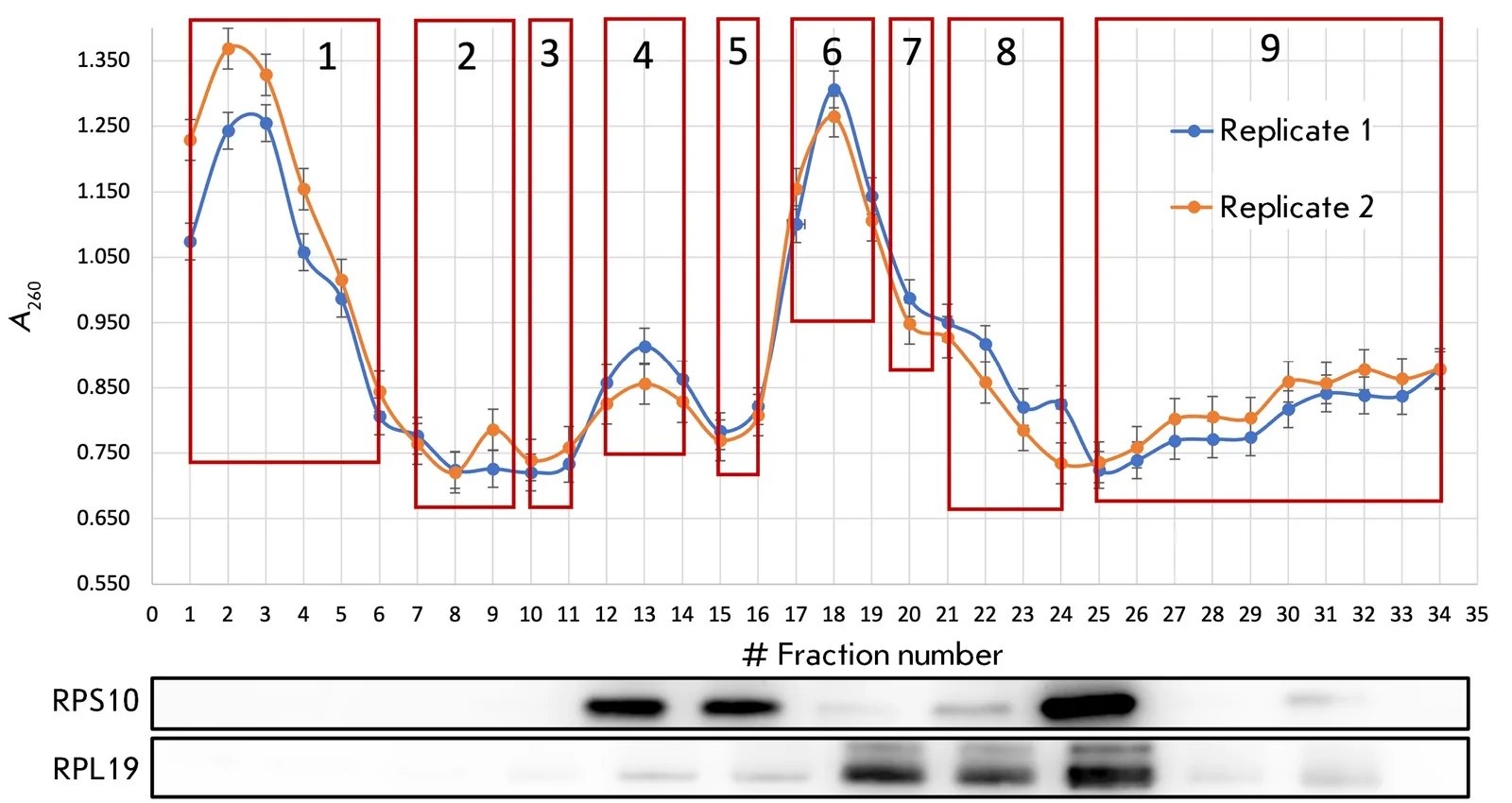
Fractionation of the nucleolar fraction after extraction with buffer 2 in a sucrose gradient and selection of pools for proteomic analysis. Upper panel: A_260_ profiles following ultracentrifugation of the nucleolar fraction in a sucrose gradient for two replicates (Replicate 1 and Replicate 2; mean ± SD of technical replicates is shown). Red boxes indicate fractions pooled (1–9) for subsequent protein extraction and mass spectrometry analysis. Lower panel: immunoblotting of pooled gradient fractions with antibodies against small and large ribosomal subunit proteins (RPS10 and RPL19)

The initial gradient fractions were pooled into nine samples denoted as 1–9. Samples 1, 2, 4, 6, 8, and 9, corresponding to different regions of the sedimentation profile and shown with red boxes in *Fig. 1*, were chosen for mass spectrometry analysis. This approach allowed us to reduce the interfraction variability and compare the protein composition of different sedimenting particles within a single experiment.

Mass spectrometry analysis of two biological replicates yielded a pooled list consisting of 1,816 unique proteins (1,424 proteins in replicate 1 and 921 proteins in replicate 2), of which 529 proteins were identified in both replicates and used as a conserved set for functional interpretation. GO analysis of the Biological Process category showed that this set was substantially enriched in protein groups corresponding to different stages of ribosome biogenesis: along with the categories “ribosome assembly” and “biogenesis of the small ribosomal subunit,” enrichment was observed for the categories “ribosome biogenesis,” “rRNA processing,” and “rRNA metabolism” (*Fig. 2A*).

**Fig. 2:**
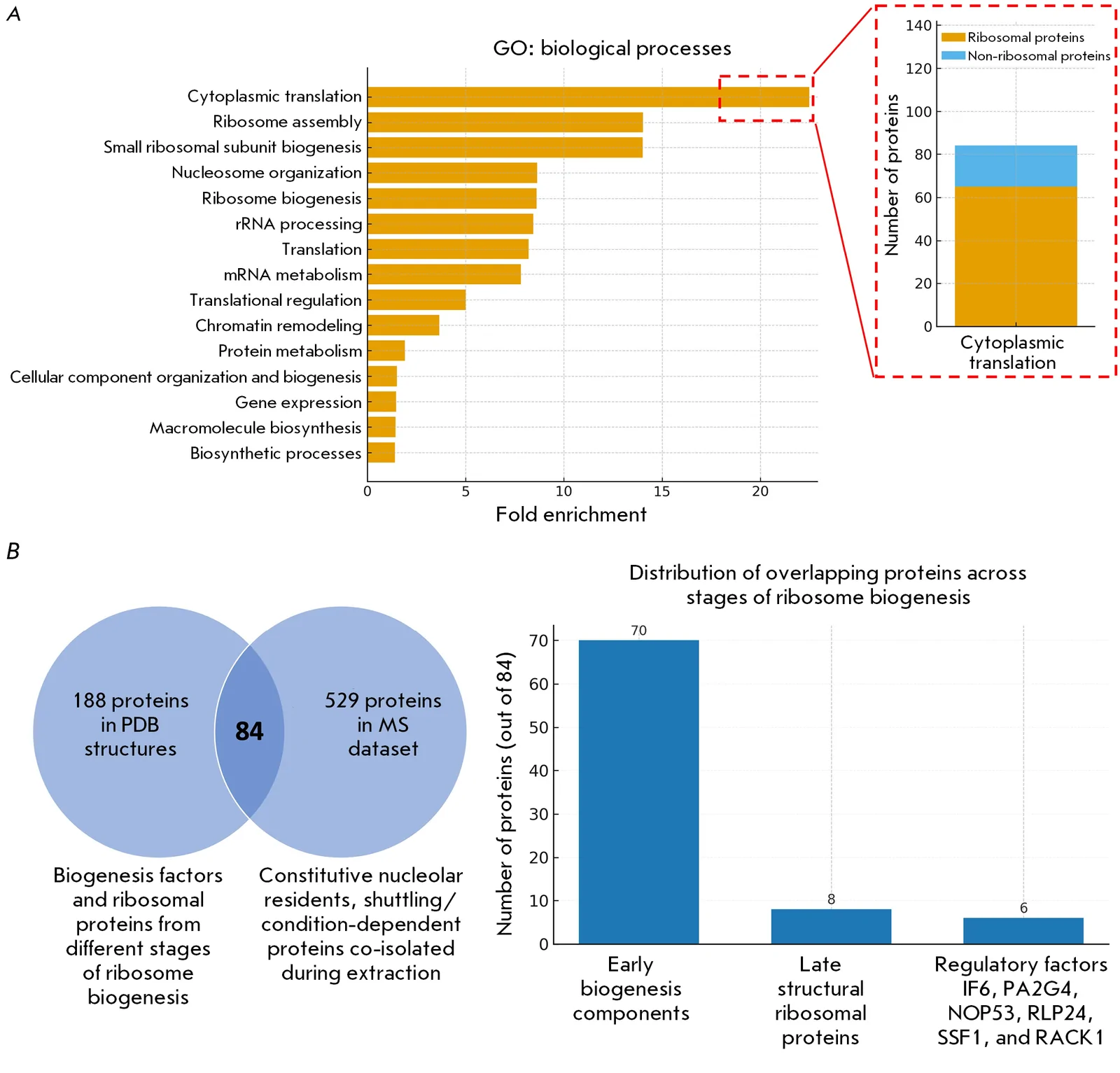
Functional annotation of mass spectrometry data using the GO analysis (Biological Process). (*A*) GO analysis of 529 proteins common to both biological replicates; (*B*) comparison of the 529-protein dataset with the list of proteins present in cryo-EM structures of human pre-ribosomal complexes deposited in the PDB database

The most significant enrichment was observed for the categories “Ribosome assembly,” “Small ribosomal subunit biogenesis,” and “Cytoplasmic translation.” An analysis of the proteins assigned to the latter category revealed that ribosomal proteins were prevalent (*Fig. 2A*). Enrichment of numerous ribosomal proteins within this set was expected, since many of them are recruited at the early stages of pre-ribosome assembly and are present in nucleolar complexes isolated during sucrose gradient fractionation. It is noteworthy that this approach aims to enrich pre-ribosomal complexes but does not preclude co-sedimentation of proteins from the nuclear and cytoplasmic compartments.

In order to perform an independent validation of the fact that the resulting set of 529 proteins adequately represents the molecular composition of pre-ribosomal particles, we cross-referenced proteins from the dataset to the list of proteins found in human pre-ribosomal complexes deposited in the PDB database (a total of 188 proteins). A total of 84 proteins overlapped between these two sets (*Fig. 2B*). The PDB entries include structures of both early and late nuclear maturation intermediates, whereas the identified nucleolar proteins are not restricted to ribosome biogenesis factors. Therefore, this overlap can be considered substantial because it includes almost half of all the biogenesis factors characterized thus far. A large subset of the remaining proteins may also participate in ribosomal subunit maturation, but this requires experimental validation. All the Biological Process categories shown in *Fig. 2A* were statistically significantly enriched; the adjusted *p*-values ranged from 9.11 × 10^-77^ (the “Translation” category mostly comprising ribosomal proteins) to 2.09 × 10^-3^ (the “Chromatin remodeling” category).

The distribution of overlapping proteins by ribosome biogenesis stage additionally showed enrichment with early components (70 out of 84), whereas the proportions of late structural ribosomal proteins (8 out of 84) and regulatory maturation/export factors (6 out of 84; IF6, PA2G4, NOP53, RLP24, SSF1, and RACK1) were smaller (*Fig. 2B*). Hence, the cross-referencing against the PDB structures support the conclusion that the isolated nucleolar fractions were enriched in early pre-ribosomal complexes.

We analyzed the combined set of 1,816 unique proteins to provide a comprehensive assessment without requiring overlap between biological replicates. Analysis of UniProt showed that, among the proteins identified in our experiment, 533 were annotated with the term “nucleolus” and 106 with the term “ribosome biogenesis.” The overlap between these annotations comprised 91 proteins (*Fig. 3*). The analyzed set was significantly enriched in all Biological Process categories shown in *Fig. 3*. The adjusted p-values ranged from 3.148 × 10^-60^ for the category “Translation,” mostly including ribosomal proteins to 2.268 × 10^-2^ for the category “Biosynthetic processes.”

**Fig. 3:**
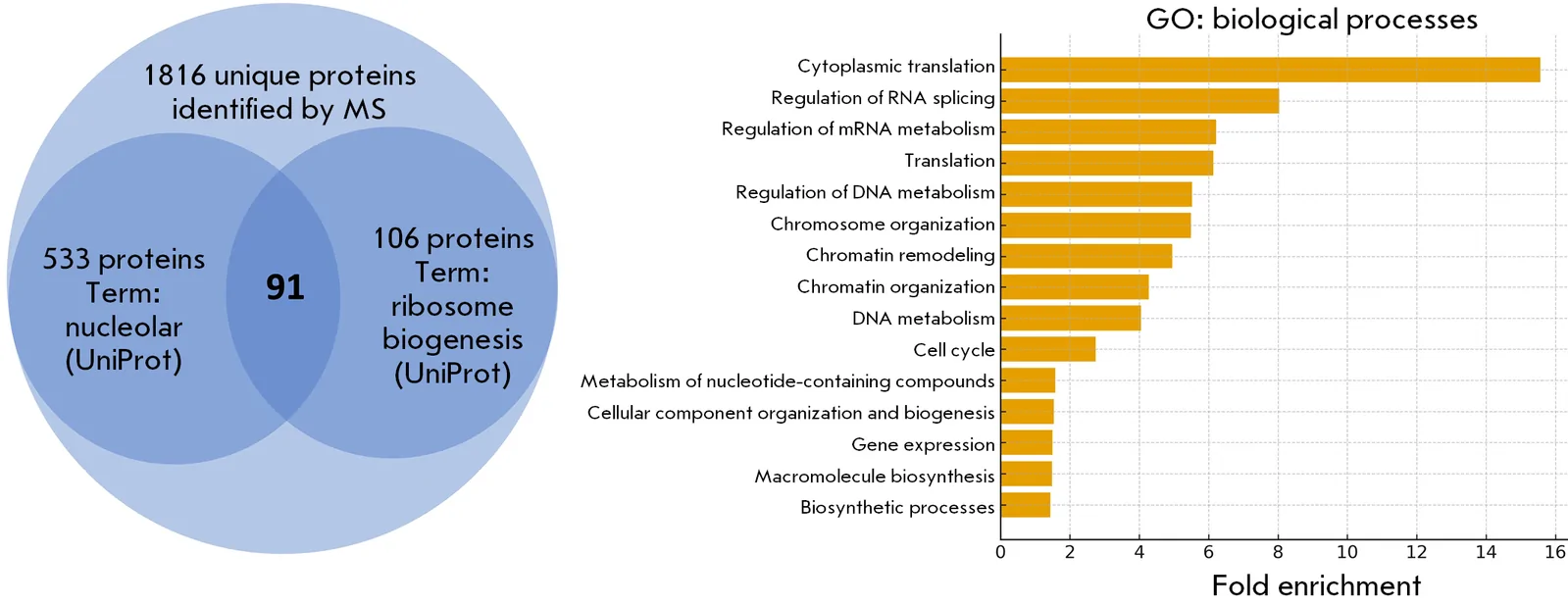
Comparison of the identified proteins with the UniProt data and functional characterization using the GO analysis

Our analysis demonstrates that most of the revealed proteins refer to the nucleolar/nuclear components and/or ribosome biogenesis factors; some proteins are simultaneously found in both functional groups. An additional GO analysis for the Biological Process category found pronounced enrichment for proteins having RNA-binding functions, including the RNA splicing regulation, mRNA metabolism, and translation (*Fig. 3*). The most abundant category (“Cytoplasmic translation”) predominantly comprises structural ribosomal proteins, which is consistent with the fact that the analyzed samples contain components of pre-ribosomal particles. Overall, the prevalence of RNA-binding and RNA-processing processes is quite expected for the nucleolar fraction enriched in pre-ribosomal complexes and shows that the nucleolus is functionally associated with both ribosome biogenesis and broader aspects of RNA metabolism. Furthermore, identification of proteins annotated by the terms “nucleolus” and/or “ribosome biogenesis” (UniProt) proves that the resulting proteomic profile is relevant for a search for novel candidate proteins that could be involved in the maturation of human ribosomal particles.

The total set (1,816 proteins) was found to contain 106 ribosome biogenesis factors (proteins involved in rRNA processing, subunit assembly, and pre-ribosome export were assigned to this category based on UniProt annotation for the keyword “ribosome biogenesis”). The sucrose-gradient distribution of pre-ribosomal complexes was assessed: the proportion of ribosome biogenesis factors increased from lighter to heavier fractions (*Fig. 4*).

**Fig. 4:**
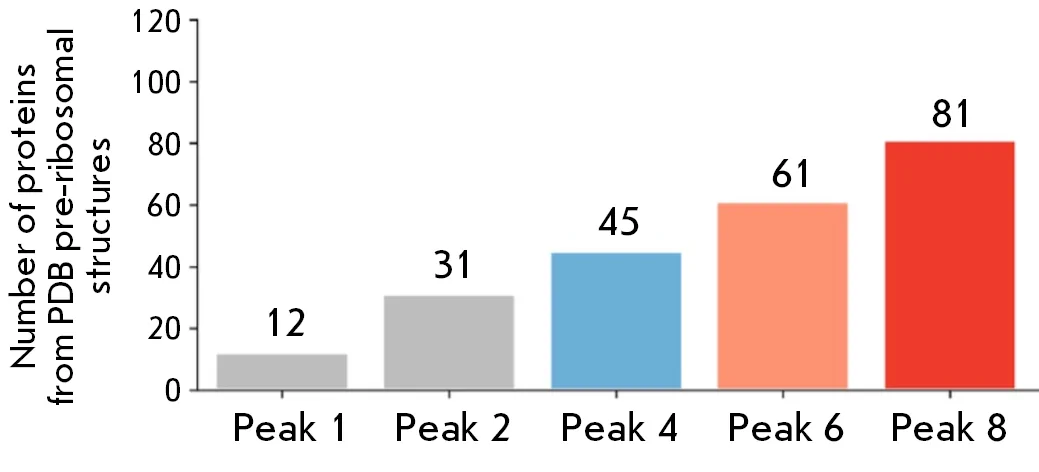
Representation of proteins from cryo-EM structures of human pre-ribosomes across gradient peaks

Proteins comprising pre-40S particles were most abundant in peak 4 (68% of all biogenesis factors) and peak 8 (63% of all biogenesis factors), which was consistent with the fact that pre-40S particles are present in light gradient fractions. Almost no proteins comprising pre-60S particles were observed in peak 4 (9%), while their content increased when proceeding toward peaks 6 (48%) and 8 (51%), demonstrating that pre-60S particles possess a higher molecular weight. The simultaneous presence of both pre-40S and pre-60S proteins in heavy fractions is likely indication that these fractions contain the earliest precursors.

## CONCLUSIONS

The proposed approach – combining sucrose gradient fractionation of the nucleolar extract with mass spectrometry analysis of the isolated pools – is an exploratory tool for investigating the protein composition of deep nucleolar fractions enriched in pre-ribosomal particles. This approach allows one to shift the focus away from the total nucleolar proteome through (i) enrichment and separation of pre-ribosomal particles; (ii) reduction of the background load from proteins from other cellular compartments; and (iii) enabling comparison of the composition of particles corresponding to different biogenesis stages. A logical next step is to functionally validate high-priority candidates using knockout/knockdown approaches and the analysis of rRNA precursors to determine their roles at specific stages of ribosome maturation. Candidates may be prioritized based on co-sedimentation with ribosome precursors and the absence of annotation as established ribosome biogenesis factors in current databases; an annotated RNA-binding function would provide additional validation. Therefore, the resulting proteomic resource and the methodological workflow lays the groundwork for the discovery and experimental verification of novel components and regulators of human ribosome biogenesis.
